# Identification of Genetic Locus Underlying Easy Dehulling in Rice-Tartary for Easy Postharvest Processing of Tartary Buckwheat

**DOI:** 10.3390/genes11040459

**Published:** 2020-04-23

**Authors:** Lijun Zhang, Mingchuan Ma, Longlong Liu

**Affiliations:** Crop Germplasms Resources Research Institute, Shanxi Academy of Agricultural Sciences, Taiyuan 030006, China; mamingchuan6@163.com (M.M.); lllong781211@sina.com (L.L.)

**Keywords:** Tartary buckwheat, easy dehulling, BSA

## Abstract

As a highly nutritious crop, Tartary buckwheat (*Fagopyrum tartaricum)* strongly adapts and grows in adverse environments and is widely grown in Asia. However, its flour contains a large proportion of the hull that adheres to the testa layer of the groats and is difficult to be removed in industrial processing. Fortunately, rice-Tartary, with the loose and non-adhering hull, provides potentiality of improving Tartary buckwheat that can dehull easily. Here, we performed high-throughput sequencing for two parents (Tartary buckwheat and rice-Tartary) and two pools (samples from the F2 population) and obtained 101 Gb raw sequencing data for further analysis. Sequencing reads were mapped to the reference genome of Tartary buckwheat, and a total of 633,256 unique SNPs and 270,181 unique indels were found in these four samples. Then, based on the Bulked Segregant Analysis (BSA), we identified a candidate genetic region, containing 45 impact SNPs/indels and 36 genes, that might underly non-adhering hull of rice-Tartary and should have value for breeding easy dehulling Tartary buckwheat.

## 1. Introduction

The genus *Fagopyrum*, belonging to the eudicot family Polygonaceae, is comprised of 27 species [1] in which two diploid species are cultivated as food crops currently: Tartary buckwheat (*Fagopyrum tataricum*) and common buckwheat (*Fagopyrum esculentum*). In recent years, Tartary buckwheat has received more attention because it outperforms common buckwheat in some respects. Tartary buckwheat has higher and more stable yield attributed to its self-low-seed abortion, compatibility, and tolerance to stress such as frost [2,3]. It is also a beneficial health crop which can produce some nutraceutical products and functional foods and shows a higher content of some nutritional components than common buckwheat, such as total vitamin B content, the flavonoid with high antioxidant activity (e.g., rutin) [4]. Rutin is known for its ability in aiding vitamin C usage, for its strong blood vessels, and for reducing high blood pressure and blood clots [5,6,7,8], and its content in Tartary buckwheat seeds is approximately 100 times (0.8–1.7%) higher than in common buckwheat (0.01%) [9]. However, the hull of Tartary buckwheat tightly adheres to the testa layer of the groats [2], and it is hard to remove hull completely. Therefore, Tartary buckwheat has to be consumed together with the flour inadvertently, which reduces the taste. Most normal mechanical dehulling in industrial processing, such as steam dehulling, is time-consuming, breaking seed, and only up to 70% of the hull can be removed [2]. Therefore, it is one of the main objectives of breeders to develop new easy dehulling Tartary buckwheat varieties to improve the taste feeling of the flour and to meet the market needs.

Rice-Tartary, also called Miqiao in China and bate Phapal in Nepal and India, is a particular Tartary buckwheat type with seeds similar to rice in several aspects including shape, size, and cooking methods [10,11] Unlike seeds of Tartary buckwheat which has three grooves on the hull, rice-Tartary has three length-wise openings or splits in its hull, which makes dehulling easier [12]. Through introgression genetic component of easy dehulling in rice-Tartary into the Tartary buckwheat genome, it is hoped to develop a new buckwheat type with loose or non-adhering hull for ease-dehull [2]. Finding the candidate genes which may relate to the construct of interest is the main task of effort in introgression, and specific genetic component of the acceptor parent (variety to be improved) could be replaced with that of donor parent (variety with desired trait performance). The easy dehulling gene in rice-Tartary has not been reported before, so identification of the genetic locus, cloning of genes, and developing linked markers related to the easy dehulling in rice-Tartary has significant implications for the breeding of Tartary buckwheat. 

The traditional gene identification method (positional- or map-based cloning) is time and labor consuming and expensive. It involves developing the genetic population with segregating phenotype of target traits, such as F2, recombinant inbreed populations, near-isogeneic lines, and so on, which need several rounds of crossing and planting [2,13,14]. Avoiding development of genetic population, genome-wide association study (GWAS) exploiting phenotype variations within natural population reduces the time required for the identification of genetic loci of potential traits of interest [15]. However, this method depends on the collection of enough germplasm samples for phenotype variation, genotype at high-density genome-wide markers that are usually generated from chip arrays, or whole genome resequencing which obviously increases the cost [15]. 

Selective genotyping reduces cost and simplifies analytical processing through a focus on selected samples with extreme phenotypes for genotyping and analyzing [16,17]. Bulked segregant analysis (BSA) [18] and DNA pooling [19] further reduce the cost significantly by bulk samples in two tails of phenotype distribution respectively and analyses them as an integrated unit. Recently, BSA has been modified to locate the target genes using positive markers, so the putative markers do not need to be validated by genotyping entire populations [17,20,21]. As a result, the cost of genotyping has been dramatically reduced from two aspects: (1) testing on fewer selective samples and (2) focusing on fewer positive markers [17,22,23]. For example, considering a population consisting of 300 samples and bulking 30 extreme samples from each tail, BSA expends only 0.6% (~2/300) of total cost required for testing all samples.

In this study, we identified a genetic locus controlling easy de-hulling in rice-Tartary by combining the BSA and high-throughput sequencing to improve Tartary buckwheat with properties of easy dehulling after harvesting. An F2 population was generated using Tartary buckwheat and rice-Tartary as parents, from which 30 rice-Tartary-like lines with easy dehulling seeds and 30 Tartary-buckwheat-like lines with seeds hard to be dehulled were selected and pooled, respectively, forming two “sequencing” lines for genotyping. The identified genetic locus, SNPs or indels, linking the gene underlying the easy dehulling in rice-Tartary, valuable for breeding Tartary buckwheat could be dehulled easily. 

## 2. Materials and Methods

### 2.1. Sample Collection and DNA Sequencing

**Figure 1:**
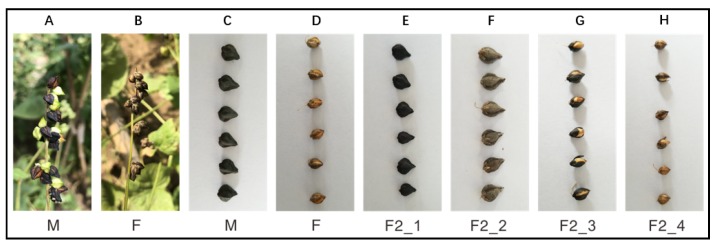
Phenotype of traits associated with the dehulling: (**A**) seed loading branch of Tartary buckwheat as male parent; (**B**) seed loading branch of rice-Tartary as female parent; (**C**) seeds of Tartary buckwheat as male parent; (**D**) seeds of rice-Tartary as female parent; (**E**) “Tartary-buckwheat-like” seeds in the F2 population; (**F**) “Tartary-buckwheat-like” seeds in the F2 population; (**G**) “rice-Tartary-like” seeds in the F2 population; and (**H**) “rice-Tartary-like” seeds in the F2 population.

In the present study, one Tartary buckwheat sample and one rice-Tartary sample were collected and used as parents to generate an F2 population. A total of 60 samples which contain 30 rice-Tartary-like lines (with easy dehulling seeds) and 30 Tartary-buckwheat-like lines (with hard dehulling seeds) were selected and pooled, respectively (Figure 1).

Genomic DNA was extracted from two parental samples and two pools, and then, four pair-end sequencing libraries with 400 bp insert size were constructed according to the factorial manual. All libraries were sequenced using the Illumina Hiseq (Illumina, San Diego, CA, USA) platform in pair-end model with 150 bp length and 150 sequencing reaction rounds. 

### 2.2. Preprocessing of Raw Sequencing Data

To reduce the effect of sequencing errors, raw sequencing reads were preprocessed to acquire high-quality data. First, reads with adapter contamination were clipped with Adapter Removal (version 2) [24]. Second, low-quality bases were cut using the sliding window method for which window size was settled to 5 bases (PERL program v.5.18.2). The dynamic window slides from reads 5′ to 3′ end and stops at the base where its quality is less than 2 or the average quality of bases in a window is less than 20. Then, bases before that terminate window sliding were preserved as continuous high-quality bases. After that, pair of reads from the same sequencing template (PE) were discarded if one or two of them were shorter than or equal to 50 bases. The remaining reads were regarded as “high-quality clean reads” and were used in further analysis.

### 2.3. Variant Analysis of SNPs and Indels

The clean reads were mapped to the genomic sequence assembly of Tartary buckwheat [25] using BWA with default parameters [26]. Output results were saved in .bam file and sorted with Picard (v.1.107) (https://www.psc.edu/index.php/user-resources/software/picard). The concordance of PE reads reflected by their mapping were checked using “FixMateInformation” package in Picard. Bias caused by duplicates, in which multiple pairs of reads mapped to exact coordinates in reference, were processed to preserve the read pair with highest mapping score and to discard others, using “MarkeDuplicates” package in Picard. The coverage depth of a base in reference was measured. Based on the mapping results, the SNPs and indels of samples were called with the OTG-snpcaller [27] and UnifiedGenotyper in GATK [28], respectively. 

### 2.4. Identification of Candidate Region by Delta SNP-Index

The SNPs were filtered according to (1) Fisher test of strand bias (FS) < 60; (2) Mapping Quality (MA) > 40; (3) Quality Depth (QD) > 4; and (4) Genotype Quality (GQ) > 20. Indels were filtered according to (1) FS ≤ 200 and (2) QD ≥ 4. SNP-index and delta SNP-index were performed using the method described by Takagi et al. [21] based on the depth information generated in variants calling. The average SNP-index of variants within a sliding window (window size of 1 MB and step size of 50 Kb) were calculated for dot plotting. Based on the results, SNPs with delta SNP-index higher than the threshold (confidence interval > 99%) were picked, and the regions flanking were regarded as the target region that might be underlying easy dehulling of rice-Tartary. The impact effect on gene function of SNPs and indels located in the target region were analyzed using ANNOVAR [29].

## 3. Results

### 3.1. DNA Sequencing for Two Parents and Two Pools 

We generated approximately 671.62 M reads with a total of 101.42 G bases from four sequencing samples: (1) KF is the rice-Tartary sample as a female parent; (2) KM is the Tartary buckwheat sample as a male parent; (3) K21 is the pool with seeds similar to rice-Tartary from F2 population; and (4) K5 is the pool with seeds similar to Tartary buckwheat from F2 population (Table 1). The number of raw reads generated from those four samples ranges from 163.00 to 175.33 M with a total length ranging from 24.61 to 26.47 Gb. After removing adaptor contamination, low-quality base, and PE with one or two reads shorter than or equal to 50 bp, 624.28 M high-quality clean reads with a total length of 91.17 Gb were contained for further analysis. The clean reads from those four samples range from 151.50 to 164.15 M with total length range from 21.98 to 23.88 Gb.

### 3.2. Mapping High-Quality Clean Reads to the Reference Genome

**Figure 2:**
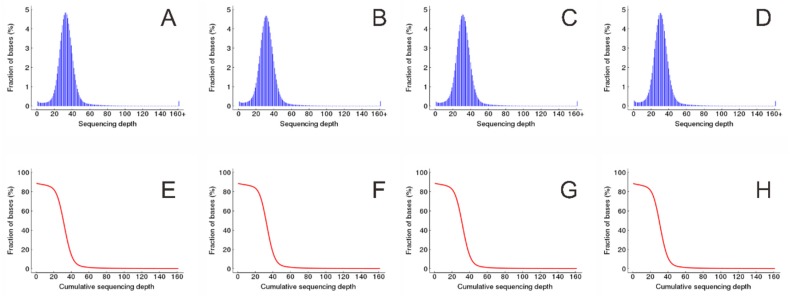
Coverage depth of reference contributed by clean reads. (**A**–**D**) Distribution of coverage depth of reference contributed by KF, K21, K5, and KM, respectively, and (**E**–**H**) cumulative distribution of coverage depth of reference contributed by KF, K21, K5, and KM, respectively.

The high-quality reads were mapped to the reference genome of Tartary buckwheat [25] using BWA [26]. There were 98.92%, 98.95%, 98.93%, and 98.95% high-quality clean reads for KF, K21, K5, and KM that could be mapped to 88.49%, 88.57%, 88.59%, and 88.39% of the genomic reference sequence, respectively (Table 2). More than ten high-quality clean reads from KF, K21, K5, and KM samples can cover 86.97%, 87.00%, 87.04%, and 86.62% of reference bases, respectively. The average coverage depth of reference contributed by high-quality clean reads from KF, K21, K5, and KM were 33.29%, 32.18%, 32.12%, and 31.40%, respectively (Figure 2 and Table 3).

### 3.3. SNPs and Indels between Samples and Reference Genome

Based on mapping results, SNPs and indels between samples and reference were called with the OTG-snpcaller [27] and UnifiedGenotyper in GATK [28], respectively. We observed 356,378, 581,386, 582,079, and 544,689 SNPs and 222,008, 261,703, 261,989, and 255,033 indels from KF, K21, K5, and KM, respectively (Table 4). The SNPs (544,689) and indels (255,033) in the KM sample (Tartary buckwheat) might be attributed to the difference between the materials used [25]. Because of the different phenotype of seed husk, more SNPs or indels were expected in KF (rice-Tatary); however, fewer SNPs (356,378) and indels (222,008) were observed in KF (rice-Tartary).

K21 and K5 showed more SNPs and indels than their parents (KF and KM). This may be explained in two aspects. First, K21 and K5 had bulked samples generated by pooling 30 extreme lines in two tails of phenotype distribution, while the KF and KM were single material. Second, progenies combine variations between their parents and the reference genome. There are some sites at which the base in reference is the same as the base in one parent but is different from the base in another parent. The genotype at those sites should be heterozygous in progenies. It is easy to infer that there should be more heterozygous genes in K21 and K5 than in parent samples KM. As expected, we do observe more heterotic sites in K21 and K5. There were 457,839 and 458,763 heterotic SNPs in K21 and K5, which were higher than the 158,972 and 306,428 in KF and KM. There were 74,637 and 74,970 heterotic indels in K21 and K5 that were also higher than the 22,871 and 44,379 in KF and KM.

### 3.4. Identification of Candidate Region Underlying Easy Dehulling in Rice-Tartary according to SNP-Index

**Figure 3:**
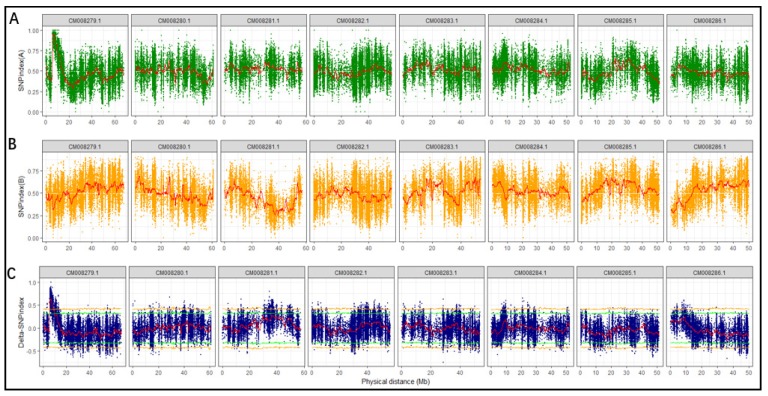
SNP-index plot: (**A**) the top panel is the plot of SNP-index for K21; (**B**) the middle panel is the plot of SNP-index for K5; (**C**) the bottom panel is the plot of delta SNP-index displaying the difference between A (top panel) and B (middle panel). The green and yellow horizontal lines represent thresholds for 95% and 99% confidence intervals, respectively.

**Figure 4:**
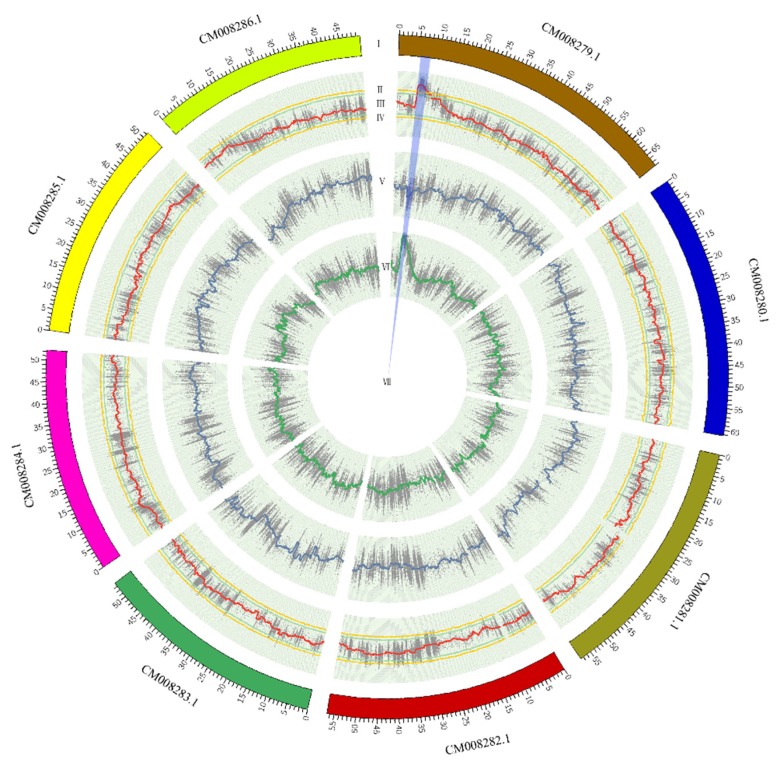
Candidate target region underlying easy dehulling of rice-Tartary: The panels from outside to inside were (Ⅰ) chromosomes; (II, III) the confidence intervals of 99% and 95% for delta SNP-index; (IV) Delta SNP-index; (V) SNP-index for K5; (VI) SNP-index for K21; and (VII) Candidate region.

SNP-index and delta SNP-index were calculated and plotted using the method described by Takagi et al. [21] which is based on depth information from SNP calling. As expected, the plot of SNP-index for K21 (rice-Tartary-like seeds) shows an obvious peak (Figure 3A) while that for K5 (Tartary Buckwheat-like seeds) presents the background (Figure 3B). The delta SNP-index, the difference between SNP-index for K5 and K21, magnified the peak at the beginning of chromosome with sequence identifier of “CM008279.1” (Figure 3C). The candidate region near the peak might contain a genetic locus underlying easy dehulling in rice-Tartary was defined which SNPs with delta SNP-index were higher than the threshold (confidence interval > 99%) (blue sector in Figure 4). The candidate region locates at the beginning of “CM008279.1”, ranging from 5,999,388 to 6,856,630 and spanning 857,243 bps. SNPs within this candidate target region were filtered according to their impact on gene function. Forty-four “nonsynonymous” SNPs and one “stop gain” SNP affecting 36 genes were observed. Seven SNPs with significant higher delta SNP-index are located in five genes with annotation of “ABC transporter-like (IPR003439) | AAA + ATPase domain (IPR003593) | ABC transporter, conserved site (IPR017871) | P-loop containing nucleoside triphosphate hydrolase (IPR027417)”; “Pentatricopeptide repeat (IPR002885) | Tetratricopeptide-like helical domain superfamily (IPR011990)”; and “Zinc finger, MIZ-type (IPR004181) | Zinc finger, RING/FYVE/PHD-type (IPR013083)”. 

## 4. Discussion and Conclusions

Tartary buckwheat, as a highly nutritious crop, has attracted increasing attention worldwide, while the hull of Tartary buckwheat is difficult to be removed [1]. Easy dehulling is important for grain processing and high-quality flour production. Here, we identified a candidate region which might be related to easy dehulling in rice-Tartary and could be used for improving postprocessing properties of Tartary buckwheat. A previous study involving crossing Tartary buckwheat with rice-Tartary and performing a progeny testing had concluded that the easy dehulling of rice-Tartary was controlled by a single recessive homozygous gene [2], but the location of the gene has not been reported yet. This might be attributed to the difficulty in crossing Tartary buckwheat with rice-Tartary to develop genetic populations, which is a fundamental basis in the traditional approach for gene mapping, location, and cloning, such as map-based cloning. 

Tartary buckwheat is the most difficult species to hybridize artificially in the genus of *Fapogyrum* because its flower is small (~2mm) and its pollen often dehisces before flower blowing [11]. Rice-Tartary has fewer flowers, and it is difficult to collect enough pollen for artificial pollination, which makes it unsuitable to be used as a male parent. Using rice-Tartary as a female parent is also a daunting task because its flower is close and the sepal is intact after cutting [30]. Therefore, the samples from the F2 population, which were generated by Tartary buckwheat and rice-Tartary, were precious for BSA. The BSA method has been successfully used to detect candidate gene for vital phenotypic characteristics in many other major crops, such as maize [31], barley [32], soybean [33,34], cucumber [35], tomato [36], and chickpea [37]. In this study, an obvious and significant peak in the SNP-index plot for K21 and the final delta SNP-index indicate a single region underlying the easy dehulling in rice-Tartary. This is consistent with results described in the previous publication suggesting a single recessive gene [2]. The SNPs strongly impacting function of genes and genes affected in candidate region encompassing the peak identified in this study have valuable significance for both developing easy dehulling varieties through trait introgression in breeding practice and gene cloning in academic research.

With the increasing interest in Tartary buckwheat products, more researchers focus on the hybridization of Tartary buckwheat. A genetic study involving seed shattering of Tartary buckwheat using intraspecific hybrids method has been reported by Fesenko [38]. In Japan, several laboratories also focus on improving the efficiency of emasculation methods such as treatments of hot water immersion, which has been utilized in crossing of rice [2]. Wang and Campbell report the hybridization between Tartary buckwheat and rice-Tartary [2]. Prospectively, the result acquired in this study, combined with the development of a simple and practicable and straightforward method of hybridization, will speed up Tartary breeding.

In conclusion, we finally identified a candidate genetic region containing 45 impact SNPs/indels and 36 associated genes through comparison and bioinformatic analysis. The candidate genetic region, SNPs/indels, and genes might underly the non-adhering hull phenotype of rice-Tartary and should have value for breeding easy dehulling Tartary buckwheat. 

## Figures and Tables

**Table 1:** Summary of data generated by sequencing.

Sample	Raw Reads		Clean High-Quality Reads						
	Read Counts	Base-Count (bp)	Reads		Bases		GC Content	Q20 Rate	Q30 Rate
			Counts	Percentage	Counts (bp)	Percentage			
KF	175,326,806	26,474,347,706	164,150,520	93.63%	23,881,470,617	90.21%	37.73%	98.77%	95.68%
K21	167,552,432	25,300,417,232	157,245,508	93.85%	22,887,256,307	90.46%	37.53%	98.79%	95.74%
K5	165,742,386	25,027,100,286	154,379,484	93.14%	22,421,430,790	89.59%	37.62%	98.74%	95.57%
KM	163,004,484	24,613,677,084	151,502,312	92.94%	21,978,979,070	89.30%	37.68%	98.72%	95.52%
Total	671,626,108	101,415,542,308	624,277,824		91,169,136,784				

KF: rice-Tartary sample as female parent; K21: pool of lines with “rice-type” seed from the F2 population; K5: pool of lines with “Tartary-type” seed from the F2 population; KM: Tartary buckwheat sample as male parent.

**Table 2:** Summary of reads mapped to reference genome.

Sample	Total Reads	All Mapped Reads		Reads with Multiple Hits		Uniquely Mapped Reads	
		Counts	Percentage	Counts	Percentages	Counts	Percentages
KF	166,011,976	164,221,550	98.92%	42,503,325	25.60%	121,718,225	73.32%
K21	158,977,495	157,302,128	98.95%	41,559,584	26.14%	115,742,544	72.80%
K5	156,090,062	154,425,747	98.93%	38,067,758	24.39%	116,357,989	74.55%
KM	153,020,562	151,421,206	98.95%	39,323,846	25.70%	112,097,360	73.26%

**Table 3:** Summary of reference bases with different coverage depth.

Sample	Average Depth	Percentage of Reference Bases with Different Coverage Depth		
		Coverage Depth ≥ 1	Coverage Depth ≥ 4	Coverage Depth ≥ 10
KF	33.29	88.49%	87.90%	86.97%
K21	32.18	88.57%	88.00%	87.00%
K5	32.12	88.59%	88.04%	87.04%
KM	31.40	88.39%	87.72%	86.62%

**Table 4:** Summary of SNPs and indel between sample and reference.

Sample	SNP			Indel		
	Total	Heterozygous	Homozygous	Total	Heterozygous	Homozygous
KF	356,378	158,972	197,406	222,008	22,871	199,137
K21	581,386	457,839	123,547	261,703	74,637	187,066
K5	582,079	458,763	123,316	261,989	74,970	187,019
KM	544,689	306,428	238,261	255,033	44,379	210,654
Union	633,256	543,128	352,990	270,181	89,724	230,763

Union is the total number of unique SNPs found in all samples.
